# Development of a cost-effective compact diode-laser-based photoacoustic sensing instrument for breast tissue diagnosis

**DOI:** 10.1117/1.JBO.29.1.017002

**Published:** 2024-01-11

**Authors:** Suhel Khan, Sramana Mukhopadhyay, Srivathsan Vasudevan, Garima Goel, Deepti Joshi, Neelkamal Kapoor, Saikat Das

**Affiliations:** a Indian Institute of Technology Indore, Department of Electrical Engineering, Simrol, Madhya Pradesh, India; b All India Institute of Medical Science Bhopal, Department of Pathology and Lab Medicine, Bhopal, Madhya Pradesh, India; c All India Institute of Medical Science Bhopal, Department of Radiotherapy, Bhopal, Madhya Pradesh, India

**Keywords:** diode laser, optical casing, photoacoustic sensing, quantitative information, spectral response, breast tissue diagnosis

## Abstract

**Significance:**

The photoacoustic (PA) technique, a noninvasive pump–probe technique, has found interesting applications in biomedical tissue diagnosis over the last decade. To take it a step further to clinical applications, the PA technique needs to be designed as an instrument focusing on a compact design, reducing the cost, and quickly providing a quantitative diagnosis.

**Aim:**

This work presents a design and characterization of a cost-effective, compact PA sensing instrument for biomedical tissue diagnosis.

**Approach:**

A compact laser diode case design is developed to house several laser diodes for PA excitation, and a pulsed current supply unit is also developed in-house to power the laser diodes to generate a 25 ns current pulse at a frequency of 20 kHz. After PA experimental data acquisition, the signal’s frequency spectra were calculated to characterize the tissue quantitatively and correlated with their mechanobiological properties.

**Results:**

The corresponding dominant frequency peak in the PA spectral response (PASR) study was low in the fibrofatty normal breast tissue 0.26±0.03  MHz, compared to the dominant frequency peak of 1.60±0.016  MHz in the fibrocystic disease tissue, which had increased glandular and stromal elements, thereby increased tissue density. The histopathological findings correlated with the PASR results, and the fibrocystic breast disease tissue exhibited a higher dominant frequency peak and energy compared to the normal breast tissue.

**Conclusions:**

We experimented with an *in vitro* PASR study of fibrocystic human breast tissues and successfully differentiated different tissue types using quantitative spectral parameters peak frequency, mean frequency, and spectral energy. This gives the potential to take this technique further for cost-effective and quick clinical applications.

## 1 Introduction

Fibrocystic changes in the breast can result in breast pain and palpable cystic masses, which need an accurate clinical diagnosis to differentiate from any neoplastic lesion. Additionally, fibrocystic changes are often noted in the peritumoral breast parenchyma in surgical specimens resected for malignancy. The gross appearance of the fibrotic areas may mimic a tumor during sampling of tissue for histopathological examination by the surgical pathologist.^1^^,^^2^ For clinical diagnosis of breast diseases, ultrasound and mammogram are the most common diagnostic modalities, followed by fine needle aspiration cytological examination.^3^ However, often the accuracy of these imaging modalities is not sufficient. Fine needle aspiration is limited by sampling issues and may often warrant a further invasive procedure such as a core needle biopsy or even an open surgical biopsy followed by a histopathological examination to avoid missing a malignant tumor and arriving at a definite diagnosis.^4^ Hence, there is a need to look for an alternative fast screening technique that provides more insight into the disease diagnosis for clinicians and may also have additional benefits in providing cues for accurate sampling of pathological breast tissue.

The photoacoustic (PA) diagnostic technique is a pump–probe technique that has evinced widespread interest among biomedical scientists in the past decade.^5^ Briefly, the principle of PAs is that it uses a pump–probe methodology to excite the biological sample. Generally, the excitation is performed by an Nd:YAG-based nanosecond pulsed laser. Upon excitation, there is a temperature excursion that is dissipated nonradiatively through ultrasound waves. An ultrasound transducer, the probe, detects the generated acoustic waves upon temperature excursion. This time domain signal is called the PA signal, which usually captures the signature of the sample through its optical absorption and tissue density. These parameters can be very well characterized through the acoustic spectra of the signal, which can be obtained through Fourier transform.^6^

On the other side, the PA signal’s amplitude represents the optical absorption of the sample, and the time delay is used to determine the distance of the biological sample. These two features reconstruct tomographic images from the acquired PA signals, called PA tomography (PAT). While PAT has shown several applications,^7^^,^^8^ it still faces several challenges for clinical applications. The primary reason is the use of an industry laser (Nd:YAG pulsed laser) at lower levels of energy for PA-based biomedical applications. The energy is kept low so that noninvasive applications can be performed within the limit of the thermal confinement of the sample. Rascevska et al.^9^ used a conventional Nd:YAG laser-based excitation system. A prototype hand-held PA imaging probe was designed using a fiber bundle. These conventional components are not portable and cost-effective solutions. Goh et al.^10^ also used a conventional bulky PA imaging system. Nyayapathi et al.^11^ and Lin et al.^12^ presented an outlook on different tomographic imaging systems containing planar, hemispherical, cylindrical imaging systems, etc. The major challenges of using these conventional lasers are that they are bulky, immobile, and require specialized training. There is a strong need for a compact, portable, and cost-effective PA excitation system.

The excitation part of the PA technique has explored including the utilization of different forms of light excitation using different methods, such as light-emitting diodes (LEDs), pulsed laser diodes (PLDs), and continuous wave laser diodes with an acousto-optic modulator.^13^ Zhu et al.^14^ used LED-based laser irradiation for human eye cancer diagnosis. They used LEDs with wavelengths ranging from 400 to 900 nm, but the energy limitation and scattering behavior are its main drawbacks. Daoudi et al.^15^ developed a PAT probe using a laser diode stack provided by Quantel (Paris, France). The wavelength used is 805 nm, making the probe suitable for specific applications with optical absorption in this range. Wang et al.^16^ attempted a PA microscopy system using a PLD. The wavelength used is 905 nm with a pulse repetition rate of 1 kHz and a pulse width of 124 ns. This experimental setup can image two tubes filled with rat blood. Zeng et al.^17^ attempted an optical resolution photoacoustic microscopy (OR-PAM) system consisting of a PLD-based light source excitation. The wavelength of the laser diode was 405±5  nm with a 174 ns pulse width with a 1 kHz repetition rate. All the methods discussed above show promising results, but none of them could reach the clinical benches.

This work aims to develop a quick screening tool using the PA sensing method. The tool should be a compact and cost-effective one so that it can be performed in remote areas, inaccessible to medical facilities. The first part of the development highlights the design of a laser diode casing system that can provide a cost-effective laser-diode-based PA excitation. After acquiring the generated PA signal, the PA spectral response (PASR) information is extracted from the acquired time-domain PA signals to analyze and differentiate biomedical tissue samples. This study aims to investigate the ability of the PASR technique to differentiate the heterogeneity of tissue properties of breast parenchyma showing fibrocystic changes compared to the normal fatty tissue of the breast and the utility of the PA technique in diagnosing fibrocystic breast disease.

## 2 Materials and Methods

### 2.1 Sample Preparation

A biological study was conducted in *in vitro* breast tissue samples following approval by the Institutional Human Ethics Committee (IHEC-LOP/2023/IL081). Archived formalin-fixed breast tissue samples of cases where the histopathology report showed the presence of fibrocystic change were retrieved from the archival section of the surgical pathology laboratory. The remaining breast tissue of the selected specimen was grossly examined for the presence of fibrotic areas and cystic lesions, and 10  mm×10  mm×5  mm breast tissue bits were sampled for the *in vitro* PASR experiment. A total of eight such samples (normal and diseased) from four different patients were collected with due approval.

### 2.2 PLD-Based PASR Sensing Instrument Configuration

**Fig. 1:**
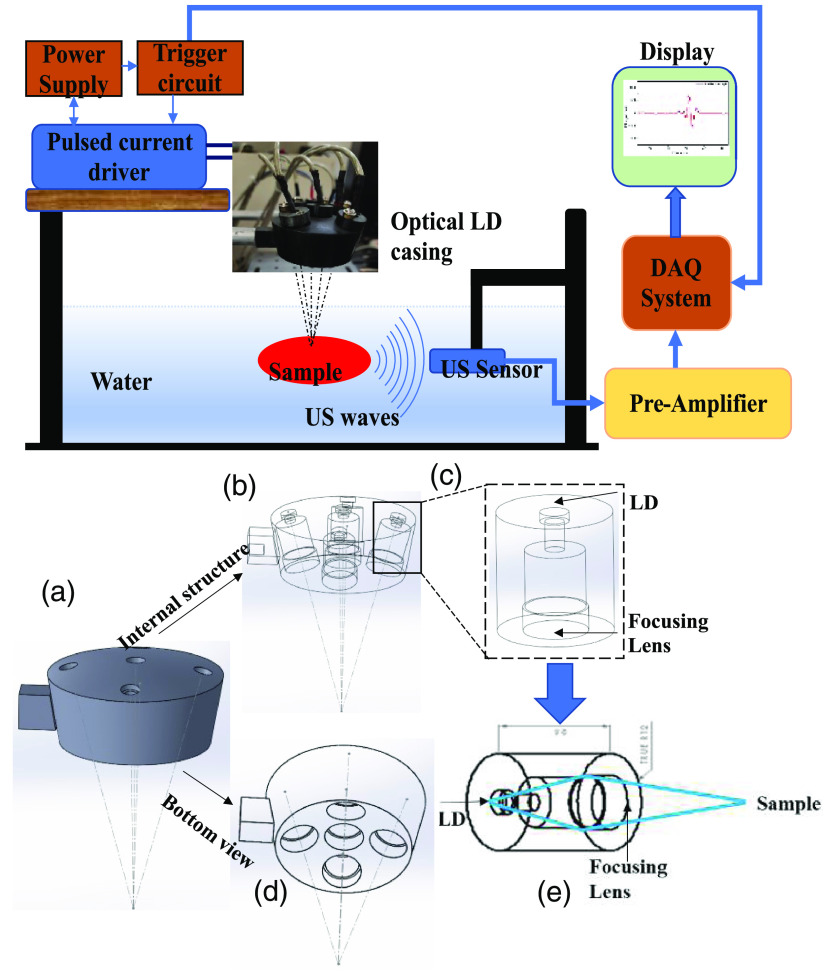
Proposed PLD-PASR-based sensing instrument and schematic design of a novel optical casing for multiple laser diodes: (a) side view, (b) transparent side view with internal assembly, (c) laser diode holder with lens assembly, (d) bottom view of the optical casing, and (e) detailed side view of laser propagation. LD, laser diode.

The first part of the experimental development is to design a system that can accommodate multiple low-cost laser diodes. Therefore, an experimental sensing instrument PLD-based PASR was developed, and the schematic of a laser diode casing is shown in Fig. 1. The casing can house five current-controlled laser diodes (PLPT5-447KA, wavelength = 447 nm, and optical power = 3.4 W). There are two reasons for choosing this laser diode (wavelength = 447 nm and optical power =3.2 W). The first one is that the wavelength is very close to 532 nm (which is the wavelength used for conventional PA experiments as the hemoglobin absorber has high optical absorption at 532 nm), and it could be used for correlation and validation with conventional PA technique. The second reason is that it offers sufficient optical power for tissue excitation in a pulsed mode, which is required for the proposed PA experiments. The customized pulsed current supply unit provides the nanosecond current pulses required for these laser diodes. The pulsed laser excitation system with single and multiple laser diode placements is shown in an optical casing in Figs. 1(a)–1(e). The optical casing was designed to hold these laser diodes in multiple axes so that all the laser diode output should be merged at a single point. This increases the intensity of the light irradiating the tissue sample.

#### 2.2.1 Pulsed current supply unit

**Fig. 2:**
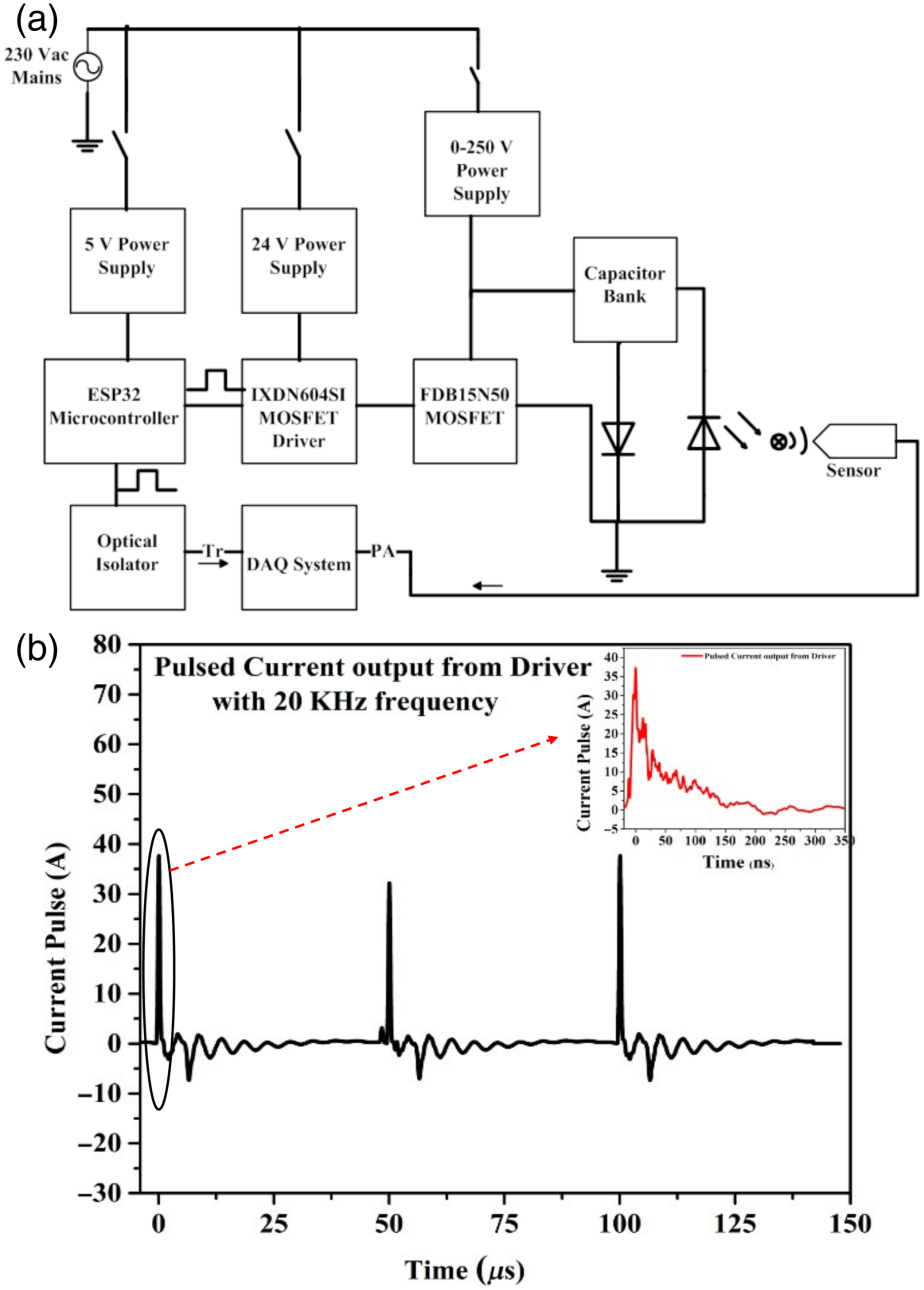
(a) Schematic design of the pulsed current supply for laser diodes. (b) Pulsed current output from the driver circuit.

The pulsed current supply unit contains a high voltage supply (0 to 250 V), a constant 24 V supply, and a microcontroller-based pulse generator. The pulsed current unit is based on a capacitor charging principle.^18^ The capacitor gets discharged for a very short period with the help of a MOSFET-FDB14N30 (MOSFET = metal-oxide-semiconductor field effect transistor) switch and delivers a high current pulse of up to 40 A to the multiple laser diodes. Figure 2(a) shows the different blocks involved in the power supply design. The design contains an ESP32 microcontroller, a MOSFET driver (IXDN604SI), and a power MOSFET. The microcontroller is programmed to generate pulses of 20 kHz frequency with a 7% duty cycle and is given to the MOSFET driver to produce a 24 V pulse signal to drive the power MOSFET. Capacitor bank (R1 to R9 and C1 to C9) is being charged by a high voltage supply of (0 to 250) V in the off condition of the MOSFET. Whenever the MOSFET driver’s pulse comes, MOSFET switches ON, and the charged capacitors are discharged immediately on the laser diode in the form of a high current pulse to produce lasing.

Pulsed current supply output has been investigated experimentally using a 100mΩ resistor in series. Figure 2(b) is an experimental characterization result of the current pulses produced from the custom-built nanosecond current supply unit. The result shows the consistency of the generated current pulses of 38±0.5 A at a frequency of 20 kHz, and the inset proves that the current pulse has a 25 ns pulse width at full width at half maximum (FWHM). This current pulse will be supplied to the laser diode for optical pulsing.

### 2.3 Characterization of the Laser Diode Casing

**Fig. 3:**
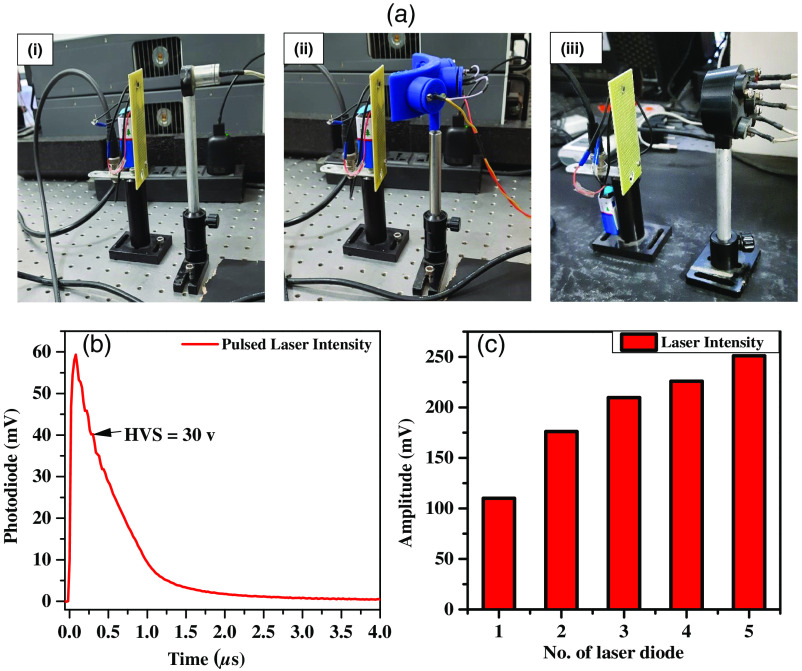
(a) Intensity measurement of the diode laser system using: (i) one, (ii) three, and (iii) five laser diodes. (b) Pulsed laser intensity measurement. (c) Peak amplitude of laser intensity of laser diodes output.

A photodiode (PD) system has been used to characterize the laser diode’s optical power. Laser diode-based excitation system characterization has been done with respect to variable power supply, output intensity measurement, and varying the number of laser diodes. Since the laser diode is a low-energy diode compared to conventional Nd:YAG laser, multiple laser diodes were necessary to be focused onto a single spot to increase laser energy at the tissue target spot. The experimental setup is shown in Fig. 3(a). Laser intensity and pulse width measurements were conducted using a custom high-speed PD-based detector and a diode laser with single and multiple laser diodes. In Fig. 3(b), a 60 mV pulsed laser intensity was observed with a 30 V input voltage to the current supply unit and stored a pulse width of around 500 ns. Figure 3(c) illustrates changes in measured pulsed intensity with varying numbers of laser diodes. This is crucial for understanding light intensity for practical applications, showing that activating multiple laser diodes consistently enhances laser intensity.

### 2.4 Frequency-Domain PA Signal Modeling and Correlation With Mechanobiological Properties

The focus of the PA sensing technique is to explore the mechanobiological characteristics of biological tissues, including factors such as elasticity and density. A standard quantitative parameter to investigate the elastic properties of biomedical samples is bulk modulus (B),^19^ which represents the tissue’s resistance to uniform volumetric compression. This equation can represent the relationship as follows:^6^

$$B=\rho_{o}\upsilon_{o}^{2},$$ (1)

where B = bulk modulus, $\upsilon_{o}$ = sound speed, and $\rho_{o}$ = tissue density. The bulk modulus calculation for various tissues in Eq. (1), along with sound speed and density, is a physical standard.^20^ For PA experiment by modeling the irradiation of a viscoelastic point object with a laser pulse, the generation and propagation of the resulting time-domain PA wave can be described as

$$\frac{\partial^{2}}{\partial t^{2}}p(t)+a^{2}\frac{\left(\xi +\frac{4}{3}\eta_{e}\right)}{d}\frac{\partial }{\partial t}p(t)+a^{2}V_{s}^{2}p(t)=\Gamma \frac{\partial }{\partial t}H(t),$$ (2)

where a represents the propagation phase constant, d signifies tissue density, ξ denotes bulk viscosity, $\eta_{e}$ represents shear viscosity, and $V_{s}$ stands for the acoustic velocity of the tissue. The Grüneisen constant Γ is defined as $\Gamma =\frac{\beta V_{s}^{2}}{C_{p}}$, where β denotes the thermal expansion coefficient and $C_{p}$ is the constant pressure heat capacity per unit mass. The function H(t) accounts for the heating induced by laser irradiation [Eq. (2)]. The damped oscillations of the PA signal, resulting from sample viscosity, serve to characterize the mechanical properties of the sample.

A comprehensive examination of PA wave generation and subsequent propagation is covered in the literature,^21^ aligning well with the experimental findings. A comparison is drawn between the PA equation and a mass-spring-damper model, enhancing our understanding of PA generation. The mass-spring-damper system is mathematically expressed as^21^

$$\frac{\partial^{2}}{\partial t^{2}}x(t)+\frac{b}{m}\frac{\partial }{\partial t}x(t)+\frac{k}{m}x(t)=\frac{F(t)}{m},$$ (3)

where m, k, and b denote the mass, spring constant, and damping coefficient, respectively. The force exerted on the mass is denoted as F(t), leading to a displacement x(t). As Eq. (3) bears similarities to Eq. (2), a comparison can be drawn, equating the parameters of the PA wave to m, k, and b, as shown in the following equations:

$$\zeta =\frac{b}{2m}=\frac{1}{2}a^{2}(\frac{\xi +\frac{4}{3}\eta_{e}}{d}),$$ (4)

$$\omega_{o}^{2}=\frac{k}{m}=a^{2}V_{s}^{2},$$ (5)

$$\omega_{d}=\sqrt{\omega_{o}^{2}-\zeta^{2}}.$$ (6)

As the PA effect involves expansion and contraction due to transient laser-induced heating, the resulting PA wave generally follows an underdamped oscillatory pattern, displaying both positive and negative peaks. By solving Eq. (3) for an underdamped system and subsequently substituting the PA wave parameters from Eq. (2), the resulting oscillating PA signal can be obtained as

$$p(t)=A_{d}e^{-\frac{1}{2}a^{2}\frac{\left(\xi +\frac{4}{3}\eta_{e}\right)}{d}t}\;\cos(\sqrt{a^{2}V_{s}^{2}-{\left(\frac{1}{2}a^{2}\frac{\left(\xi +\frac{4}{3}\eta_{e}\right)}{d}\right)}^{2}}t-\frac{\pi }{2}).$$ (7)

The value of the constant $A_{d}$ is established through the initial state of the system, which involves uniform optical illumination within the region of interest. This adjustment aligns the model more closely with actual experimental conditions. The resulting pressure wave comprises two components: a sinusoidal term and an exponentially decaying term, collectively giving rise to an underdamped oscillatory pattern. These components encapsulate the sample’s specific parameters, rendering the generated pressure wave a genuine representation of the sample’s nature. To effectively leverage this PA wave quantitatively, our experiments extract spectral information from these signals in the time domain. Equation (7) can be further simplified in a subsequent manner:

$$p(t)=A_{d}e^{-\alpha t}\;\sin\;\omega_{o}tu(t),$$ (8)

where $\alpha =\frac{1}{2}a^{2}\frac{\left(\xi +\frac{4}{3}\eta_{e}\right)}{d}$, $\omega_{o}=\sqrt{a^{2}V_{s}^{2}-(\frac{1}{2}a^{2}\frac{\left(\xi +\frac{4}{3}\eta_{e}\right)}{d}{)}^{2}}$, and u(t) is a unit step function. Apply Fourier transform to Eq. (8) would yield

$$P(\omega )=A_{d}\frac{\omega_{o}}{\omega_{o}^{2}+{\left(\alpha +j\omega \right)}^{2}}.$$ (9)

Equation (9) and $\omega_{o}$ make it evident that the frequency components of the generated PA wave are intricately tied to the viscosity, density, and other mechanical attributes of the sample. Under the assumption of a lossless medium with negligible impedance mismatch, the transmission of this generated PA wave adheres to the wave equation. This theoretical understanding was both confirmed through an experimental investigation involving black rubber and validated via simulation studies, as depicted in Refs. 22 and 23. These simulation studies revealed that an increase in density leads to a corresponding rise in sound speed, thereby causing a shift in the frequency spectra toward the higher frequency range.

### 2.5 Extraction of the PASR Spectral Parameters from Frequency Spectra to Differentiate Biological Tissues

The PASR and its analysis offer a promising sensing method to differentiate samples based on their mechanobiological properties.^23^^,^^24^ The time-domain PA signal of the different tissue samples was acquired through an ultrasound transducer (OLYMPUS parametric NDT-V303 model, 3.5 MHz center frequency with 8 MHz bandwidth). Time averaging has been implemented to improve the signal-to-noise ratio (SNR) of the acquired signal. Therefore, the acquired signal was averaged 4000 times before storing the PA signal. The pulse repetition rate of the developed diode laser is 20 kHz, which is much higher compared to the conventional Nd:YAG laser repetition rate (10 to 20 Hz). Therefore 4000 times averaging takes only 0.2 s. Focus has been kept to minimize the amount of laser energy irradiated on the sample, and hence the experiment required 4000 times averaging to improve the signal strength. Then, spectral response was obtained by performing the fast Fourier transform (FFT) of the time-domain PA signal. The obtained spectral parameters from the FFT spectrum are peak frequency (PF; $f_{p}$), mean frequency (MF; $f_{m}$), and spectral energy ($E_{s}$), which are used for quantitative tissue characterization. These parameters were obtained through the following equations:

$$E_{s}=\overset{\infty }{\underset{n=-\infty }{\sum }}{\left[X(n)\right]}^{2},$$ (10)

$$f_{p}=f_{\max[X(n)]},$$ (11)

$$f_{m}=\frac{\sum_{j=1}^{M}f_{j}P_{j}}{\sum_{j=1}^{M}P_{j}},$$ (12)

where $f_{j}$ = frequency of PA signal, $f_{p}$ = peak frequency, $f_{m}$ = mean frequency, $P_{j}$ = power-spectral magnitude, M = length of the bin, and X(n) = frequency spectrum of the PA signal, respectively. The spectral energy $E_{s}$ was calculated initially with a 0 to 10 MHz frequency range corresponding to the bandwidth of the ultrasound transducer. The effect of the low-frequency region (0 to 1 MHz) and high-frequency region (1 to 10 MHz) at the spectral energy was investigated. The spectral energy at low-frequency region $E_{L}$ and high-frequency region $E_{H}$ was normalized with the $E_{s}$, then resultant relative energies $E_{LS}$ and $E_{HS}$ were used to monitor the quantitative change at a specific frequency region, which can provide crucial information for tissue characterization.^6^^,^^22^

## 3 Results

### 3.1 Implementation of the PLD-PASR Sensing Instrument for PA Signal Acquisition

**Fig. 4:**
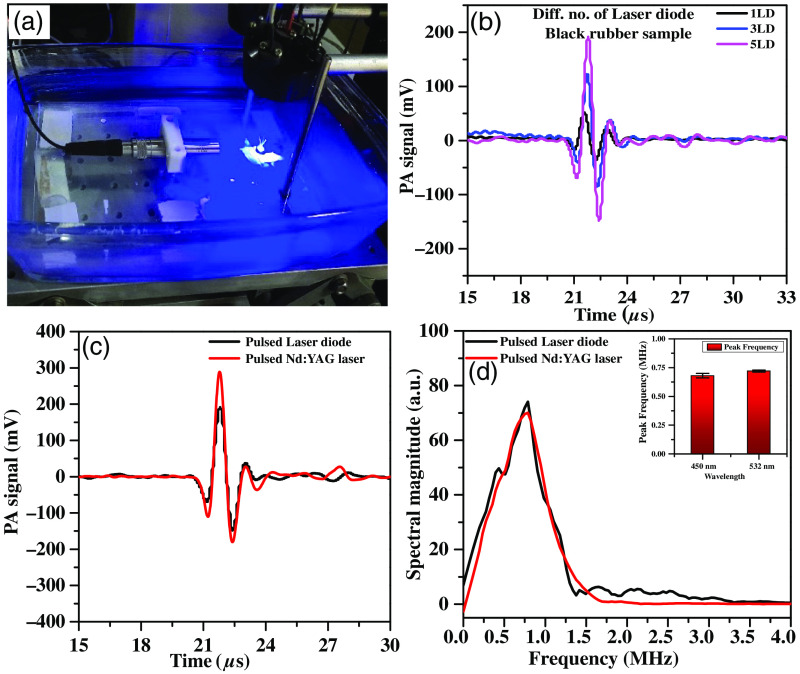
(a) Experimental setup with the casing-based PA sensing instrument. (b) Time-domain PA signal with multiple numbers of laser diodes, correlation between developed pulsed diode laser and conventional Nd:YAG laser. (c) Time-domain PA signal of a black rubber sample and (d) its spectral response.

A black rubber sample was excited with a developed diode laser’s pulsed energy. The resulting time-domain PA signal was captured using a ultrasound (US) transducer [Fig. 4(a)]. By adjusting the number of laser diodes (from one to five), pulsed energy and optical absorption were varied. Notably, Fig. 4(b) displays an ascending trend in time-domain PA signal amplitude for one, three, and five diodes, directly linking pulse energy to signal strength. This setup successfully concentrated all five laser diodes on one spot, heightening the PA signal. The accuracy of the developed diode laser was correlated with a conventional Nd:YAG laser. Figures 4(c) and 4(d) show time-domain and frequency-domain PA signals from both lasers. The diode laser’s frequency response [Fig. 4(d)] aligns with the Nd:YAG laser, validating its suitability for precise sample property investigation through the PASR technique (Table 1).

**Table 1:** Comparative study between conventional and diode laser using the PASR on black rubber sample.

Laser	PA signal^a^ (mV)	PF^b^ (MHz)	MF (MHz)
Diode laser	370 ± 0.002	0.78 ± 0.042	0.67 ± 0.021
Nd:YAG laser	490 ± 0.052	0.78 ± 0.012	0.67 ± 0.011

a Peak–peak PA amplitude.

b Dominant frequency.

### 3.2 PASR Study on Agarose Sample to Study the Density Effect

**Fig. 5:**
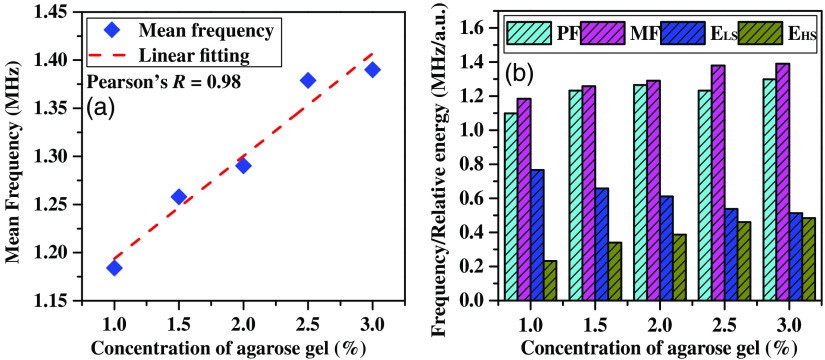
(a) Concentration of agarose gel versus the PF obtained from PASR. (b) Characterization of the agarose sample concentration using spectral parameters.

To understand the density effect on PASR parameters, five identical size agarose samples were prepared with 0.015 to 0.075  g/mL of agarose concentration and kept black ink with constant concentration (4 mL). Figure 5(a) illustrates the prompt increase in the FFT-derived MF with the enhancement in agarose concentration in tissue gels. For example, a 0.5% increase in agarose concentration instigates an enhancement of up to 7% in the MF of the PASR. Figure 5(a) also delineates a highly linear (Pearson’s R=0.98) relation between PASR-MF and gel density and, thereby, elasticity. In addition to the MF, the PF and spectral energy were also characterized ($E_{s}$) to differentiate the tissue types. Therefore, Fig. 5(b) shows the increase in MF by 7% and in PF by 12% per 0.5% increase in agarose concentration. Additionally, two frequency bandwidths were defined as 0 to 1 MHz and 1 to 10 MHz, respectively, for normalized relative spectral energy $E_{LS}$ and $E_{HS}$ conclusion, respectively. $E_{LS}$ has shown a decrease by 14.1% while increasing the concentration of the agarose. This confirms the spectral energy contribution at low-frequency regions reduces as the density of the sample increases. However, the spectral energy at the high-frequency region was increased by 47% per 0.5% increase in agarose concentration.

This study clearly says that the spectral shift and relative spectral energy can be correlated with the tissue/sample density. Therefore, the PASR can be a great tool to investigate a tissue sample’s mechanobiological properties.

### 3.3 *In Vitro* PASR Study on Breast Tissue Sample

**Fig. 6:**
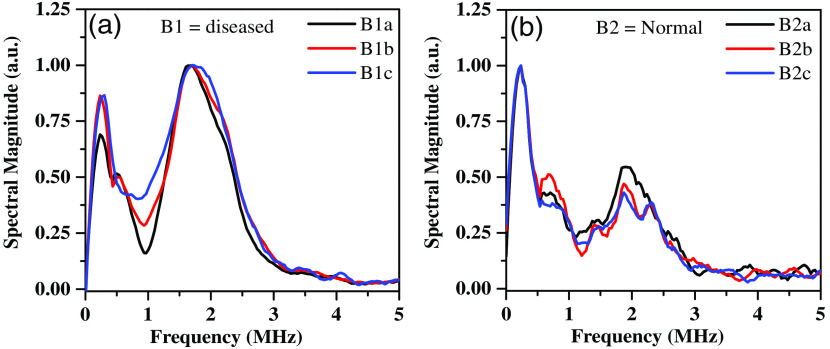
PASR response of (a) fibrocystic breast disease tissue and (b) normal breast tissue sample.

**Fig. 7:**
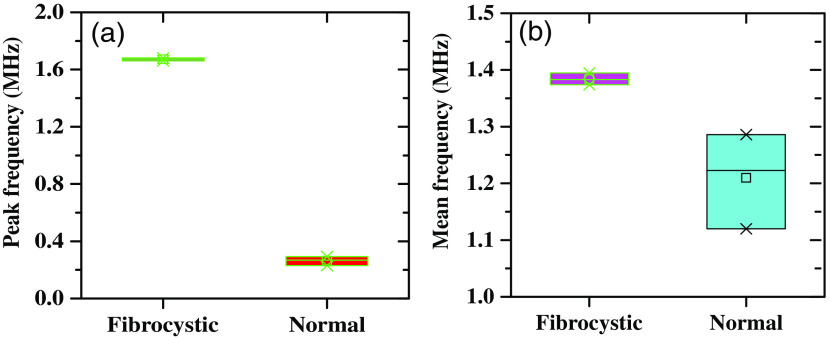
PASR spectral parameters for fibrocystic breast disease tissue and normal breast tissue (a) PF and (b) MF.

An *in vitro* experiment using the PASR technique was conducted on archived formalin-fixed breast tissue samples. The developed system investigated both the fibrocystic breast disease tissue (B1) and normal breast parenchyma (B2) samples. Ba, Bb, and Bc are three different locations in the 1  cm×1  cm size tissue sample. The time-domain PA signal was utilized to obtain corresponding frequency responses (Fig. 6) that were used to extract the spectral parameters such as PF, MF, and spectral energy. A total of eight such samples (normal and diseased) from four different patients were collected with due approval. The averaged dominant peak $f_{p}$ and MF $f_{m}$ of normal breast tissue lie between 0.26±0.005  MHz and 1.45±0.43  MHz, whereas the nontumor fibrosis breast disease tissue’s dominant frequency peak and MF are 1.66±0.23  MHz, 2.04±0.44  MHz, respectively. These frequency shifts in $f_{p}$ indicate that significant change exists in the tissue elasticity of B1 and B2 samples as well as $f_{m}$ also shows a higher signal broadening of B1 compared to B2 that indicates an increase in the tissue density as shown in Fig. 7.

In addition to dominant frequency peaks, we have also characterized the spectral energies at different frequency regions to differentiate the tissue type based on their mechanobiological properties. The importance of this characterization lies in the fact that $E_{LS}$ and $E_{HS}$ are heavily influenced by the concentration of scatterers that react to specific frequency ranges. The results in Table 2 demonstrate that T1 (fibrocystic breast disease tissue of different patients) tissue exhibits $E_{LS}$ and $E_{HS}$ values of 0.43 and 0.57, respectively, whereas T2 (breast fat of different patients) is predominantly enriched by low-frequency components ($E_{LS}=0.84$ and $E_{LS}=0.15$). These findings align with the frequency peaks result mentioned earlier, further suggesting that the proportion of spectral energy in the high-frequency range rises in relation to the bulk modulus. To check the repeatability of this technique, PA sensing experiments were performed on a total of eight such samples (normal and diseased) from four different patients and were collected with due approval. The averaged MF, PF, and spectral energy are shown with respect to all the tissues represented as T1 (disease) and T2 (normal), along with their standard deviation (Table 2). These results justify that PASR has the potential to distinguish between normal breast tissue and fibrocystic breast disease tissue.

**Table 2:** Quantitative differentiation of all breast tissue samples based on the averaged PA spectral parameters.

Tissue	PF (MHz)	MF (MHz)	Relative proportion of spectral energy	
			$E_{LS}$	$E_{HS}$
T1^a^	1.66±0.23	2.04±0.44	0.43±0.035	0.57±0.022
T2^b^	0.26±0.005	1.45±0.43	0.84±0.048	0.15±0.041

a Fibrocystic breast disease tissue.

b Normal breast parenchyma sample.

**Fig. 8:**
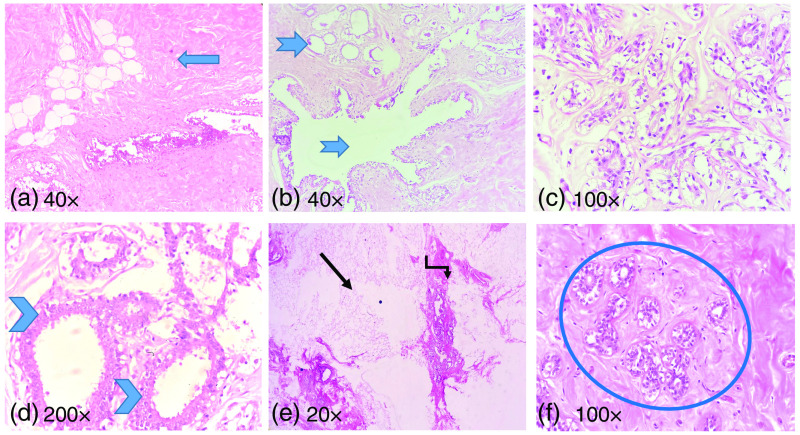
(a) Fibrocystic change in breast showing extensive stromal fibrosis (block arrow). (b) Cystic dilatation of ducts and acini (notched arrow). (c) Adenosis showing enlarged lobule with increased number of glands. (d) Mild epitheliosis of the acini showing increased cellularity (chevron). (e) Normal breast tissue with abundant fatty stroma (arrow), and terminal ductal lobular units (elbow connector arrow). (f) Normal terminal ductal lobular unit in breast parenchyma (encircled).

Following the PASR experiments of the formalin-fixed breast tissue samples, they were processed into paraffin-embedded tissue blocks and cut on a rotary microtome into 4 to 5  μm thin sections onto glass slides and stained with hematoxylin and eosin stain. The stained tissue sections were then analyzed for their histopathological features under a light microscope. The sections from B1 showed typical features of fibrocystic breast disease with areas of stromal fibrosis containing abundant extracellular collagenous matrix that cause an increase in the tissue density as shown in Fig. 8(a) and cystic dilatation of the ducts and acini of terminal ductal lobular units [Fig. 8(b)], increased glandular elements in the form of adenosis [Fig. 8(c)] and mild epitheliosis of the acini causing increased cellularity shown in Fig. 8(d) that also increase in tissue density, which verifies and correlates with the spectral parameters obtained from the PASR study.

The sections from sample B2 showed abundant fatty tissue composed of mature adipocytes [Fig. 8(e)] and only scattered terminal ductal lobular units [Fig. 8(f)], which had unremarkable histology. There was no increase in glandular or stromal elements, and only abundant fat, which refers to the comparatively lower density of the tissue. In the PASR study conducted, the corresponding dominant frequency peak was low in the fibrofatty normal breast tissue 0.26±0.03  MHz, compared to the dominant frequency peak of 1.60±0.016  MHz in the fibrocystic disease tissue, which had increased glandular and stromal elements, thereby increased tissue density. The histopathological findings correlated with the PASR results, as the fibrocystic breast disease tissue exhibited ($f_{B1}>f_{B2}$ and $E_{HSB1}>E_{HSB2}$) a higher dominant frequency peak and energy compared to the normal breast tissue.

## 4 Discussion

The paper introduces a laser diode casing designed for PA sensing applications. Results indicate that increased pulsed energy from more laser diodes enhances the PA signal amplitude. The casing effectively focuses multiple laser diodes onto one spot, boosting signal strength. This enhances the PA signal amplitude, crucial for SNR improvement. A correlation study compares the new laser diode casing-based PA system with the traditional Nd:YAG laser-based PA system, showing similar PA responses.

Later, the PASR technique also correlates the PA spectral parameter with the elastic properties of fibrocystic breast disease tissue (B1) and normal breast parenchyma (B2) tissue samples. The sound speed of the medium is directly proportional to the frequency spectra [Eq. (5)] in correlation with bulk modulus or elastic modulus, as discussed in Sec. 3.3. The contrast between normal and diseased tissue stiffness has been utilized in ultrasound elastography and magnetic resonance imaging (MRI)-guided elastography techniques.^25^^,^^26^ Optical coherence tomography has been used for microscale displacement measurements but has limited penetration depth.^27^ Based on changes in tissue elasticity, the proposed PA spectral sensing technique offers robust and predictable differentiation of fibrocystic breast tissue.

Finally, the results exhibit PASR’s potential in characterizing tissues based on dominant frequency peaks, spectral energy, and correlation with physical properties. Density-varied agarose samples confirm the relationship between elastic modulus, frequency shift, and spectral energy. Finally, an *in vitro* investigation and correlation were made between PASR findings and histopathological features using breast tissues. These findings support the potential clinical applications of PASR in diagnosing and studying various pathological conditions. Further research and advancements in PASR, including the use of advanced signal processing techniques and high-frequency transducers, could enhance its capabilities for real-time and *in vivo* studies, expanding its clinical utility. The developed in-house laser system, based on cost-effective laser diodes and a novel laser diode casing, is an initial step toward demonstrating the feasibility and potential of our approach. The future prospect is required for incorporating laser diodes with different wavelengths into the same optical casing to investigate biological tissues across a wide range of wavelengths.

## 5 Conclusion

A laser diode-based PASR sensing instrument is developed to accommodate multiple laser diodes. Optical laser diode casing and power supply unit have been characterized in terms of pulse width, laser intensity, and repeatability with multiple laser diodes. Time-domain PA signal amplitude justified the increase of the optical energy with the increased number of laser diodes. Acoustic spectral magnitude showed the developed sensing instrument’s efficacy with a conventional PA setup. Also, the PASR has been acquired to distinguish the elastic properties of the different samples. The PASR study demonstrated that fibrocystic breast disease tissue had a higher dominant frequency peak and energy compared to normal breast tissue. These findings suggest that the PLD-PASR sensing instrument could potentially serve as a noninvasive method for assessing tissue density and identifying pathological changes in breast tissue.

## Data Availability

Unrestricted code and data are available upon reasonable request to the corresponding author.
